# Changes in subcellular localisation of MI-ER1*α*, a novel oestrogen receptor-*α* interacting protein, is associated with breast cancer progression

**DOI:** 10.1038/sj.bjc.6604518

**Published:** 2008-07-29

**Authors:** P L McCarthy, F C Mercer, M W J Savicky, B A Carter, G D Paterno, L L Gillespie

**Affiliations:** 1 Division of BioMedical Sciences, Faculty of Medicine, Terry Fox Cancer Research Laboratories, Memorial University of Newfoundland, St John's, NL A1B 3V6, Canada; 2 Department of Pathology and Laboratory Medicine, Faculty of Medicine, Memorial University of Newfoundland, St John's, NL A1B 3V6, Canada

**Keywords:** nuclear localisation, MI-ER1, oestrogen receptor, LXXLL motif, breast carcinoma, immunohistochemistry

## Abstract

The oestrogen receptor-*α* (ER*α*) plays a key role in breast development and tumorigenesis and inhibiting its activity remains a prime strategy in the treatment of ER*α*-positive breast cancers. Thus, elucidation of the molecular mechanisms responsible for regulating ER*α* activity may facilitate the design of new, more effective breast cancer therapies. The MI-ER1*α* is a novel transcriptional repressor that contains an LXXLL motif for interaction with nuclear hormone receptors. We investigated the ability of MI-ER1*α* to bind to ER*α* in HEK293 and MCF-7 breast carcinoma cells, using co-immunoprecipitation assays. In both cell lines, MI-ER1*α* interacted with ER*α* in the presence and absence of oestrogen, but the interaction was stronger in the absence of ligand. Functional analysis revealed that overexpression of MI-ER1*α* in T47D breast carcinoma cells results in inhibition of oestrogen-stimulated anchorage-independent growth, suggesting that MI-ER1*α* may play a role in regulating breast carcinoma cell proliferation *in vivo*. To explore this further, we performed an immunohistochemical analysis of normal breast tissue and breast carcinoma; a total of 110 cases were examined in whole tissue sections and 771 cases were analysed in tissue microarrays. No consistent difference in the MI-ER1*α* expression level between normal breast tissue and breast carcinoma was discernible. However, there was a dramatic shift in the subcellular localisation: nuclear MI-ER1*α* was detectable in 75% of normal breast samples and in 77% of hyperplasia, but in breast carcinoma, only 51% of DCIS, 25% of ILC and 4% of IDC contained nuclear staining. This shift from nuclear to cytoplasmic localisation of MI-ER1*α* during breast cancer progression suggests that loss of nuclear MI-ER1*α* might contribute to the development of invasive breast carcinoma.

Steroid hormones, in particular oestrogens and progesterones, are crucial not only for normal growth and development of the mammary gland, but also as growth factors for the large majority of mammary carcinomas (Anderson, 2002; Clarke *et al*, 2004). Indeed, ER*α* status of breast tumours provides a powerful prognostic and predictive indicator to guide treatment regimens. Moreover, therapies that target oestrogen synthesis (oopherectomy:aromatase inhibitors) and/or that block oestrogen action on its receptor are critical for the management of hormone-dependent breast cancers (Arnedos and Smith, 2007).

In the classic pathway, the cellular response to oestrogen is initiated by hormone binding to ER*α* (Clarke *et al*, 2004; Hall and McDonnell, 2005). This high-affinity binding leads to a conformational alteration of the receptor, nuclear translocation and homodimerisation of the receptor complex. These changes permit the ER*α* complexes to bind sequence-specific oestrogen response elements located in the regulatory regions of oestrogen target genes, thus controlling their level of transcription. As with any biological pathway, this simple scheme is influenced by additional regulatory mechanisms in the cell (Ascenzi *et al*, 2006). These include phosphorylation of ER*α*, crosstalk with other signal transduction pathways, interactions with alternate receptor isoforms, like ER*β*, and binding of ER*α* to coactivator and corepressor proteins, all of which can influence the cell's transcriptional response to oestrogen. It is now clear that tumour growth in response to oestrogen and to anti-oestrogen therapies will depend upon the sum total of regulatory effects acting on ER*α*. Thus, understanding the molecular mechanisms involved in controlling ER*α* activity in tumour cells will not only be critical for the development of new markers for screening and early detection, but also for the identification of additional prognostic indicators for treatment design and novel targets for the development of more effective breast cancer therapies.

Mesoderm induction early response 1 is a novel, highly conserved transcriptional regulator (Paterno *et al*, 1997, 1998; Thorne *et al*, 2005) discovered during a screen for fibroblast growth factor response genes (Paterno *et al*, 1997). The MI-ER1 protein includes several domains common to transcriptional regulators: the N terminus contains four acidic stretches that function as an acidic activation domain (Paterno *et al*, 1997). Immediately downstream is an ELM2 domain, followed by a SANT motif; the ELM2 domain is involved in recruitment of histone deacetylase (HDAC) activity, which leads to changes in chromatin structure and results in transcriptional repression (Ding *et al*, 2003). Likewise, the MI-ER1 SANT domain functions in gene repression by interacting with Sp1 and interfering with its ability to bind to its cognate site on responsive promoters (Ding *et al*, 2004). Thus, MI-ER1 has the ability to function as both activator and repressor of gene transcription, depending on the cellular context.

Characterisation of the human *mi-er1* gene revealed that there are two major protein isoforms, MI-ER1*α* and MI-ER1*β*, which differ in their C-terminal sequence (Paterno *et al*, 2002). Of particular interest is MI-ER1*α*, as it contains in its C terminus a consensus LXXLL interaction domain characteristic of nuclear hormone receptor (NR) coregulators. Initial expression analysis indicated that *mi-er1* mRNA was differentially expressed in breast carcinoma cell lines and breast tumours (Paterno *et al*, 1998); however this study did not distinguish between the two MI-ER1 isoforms. In the current report, we examined the ability of MI-ER1*α* to interact with ER*α*, its effect on oestrogen-stimulated growth and its expression pattern in normal human breast tissue and primary breast carcinoma.

## Materials and methods

### Cell lines, plasmids and transfections

The cell lines MCF-7 and HEK293 were obtained from the American Tissue Culture Collection (Manassas, VA, USA); the T47D Tet-On cell line was purchased from Clontech (Mountain View, CA, USA). All cell lines were cultured according to the supplier's instructions. For oestrogen treatment, the media was replaced with phenol red-free media supplemented with 10% charcoal-stripped foetal bovine serum, 24 h before stimulation; 10 nM 17-*β* estradiol (E2) (Sigma) was added to the culture medium 3 h before harvesting.

The pCS3+MT containing *hmi-er1α* (Genbank: NM_001077704) has been described elsewhere (Ding *et al*, 2003). pTRE-tight-*hmi-er1α* was generated by inserting a *Bam*HI*–Xho*I fragment from pCS3+MT-*hmi-er1α* into *Bam*HI*–Sal*I cut pTRE-tight vector (Clontech). The pcDNA3 vector containing hER*α* cDNA (Genbank: NM_00125) was a gift from Dr Christine Pratt (University of Ottawa). All plasmids were sequenced to verify the junctions and the MI-ER1*α* or hER*α* sequence.

All transfections were performed as previously described (Ding *et al*, 2003) in 6-well plates, using 1.6 *μ*g of plasmid DNA. A total of 10^5^ cells per well were seeded 18 h before transfection and cells were harvested after 48 h in culture.

### Antibodies

The MI-ER1*α* antibody is a rabbit polyclonal antibody directed against a synthetic peptide representing alpha-specific sequence (amino acids 413–426); its production has been described and its specificity has been determined previously (Paterno *et al*, 2002; Thorne *et al*, 2008). Purified IgG was prepared from pre-immune or immune serum using the Melon Gel IgG purification kit (Pierce, Rockford, IL, USA) according to the manufacturer's instructions. Anti-ER*α* antibodies (D-12 and HC-20) were purchased from Santa Cruz Biotechnology Inc. (Santa Cruz, CA, USA).

### Co-immunoprecipitation and Western blot analysis

For the co-immunoprecipitation (co-IP) assays, either 1 × 10^6^ non-transfected MCF-7 cells or 2 × 10^5^ HEK293 cells co-transfected with pcDNA3-*erα* and either pCS3+MT or pCS3+MT-mier1*α* were used per sample. Cells were lysed as shown by Ding *et al* (2003) and subjected to immunoprecipitation and western blotting as shown by Ryan and Gillespie (1994). For determination of expression levels, cells were lysed directly into SDS–PAGE loading buffer and equivalent amounts of protein analysed by western blot as above.

### Establishment of stably transfected T47D cell clones and doxycycline induction

Generation of MI-ER1*α* Tet-On T47D cell clones was done as described by Ding *et al* (2004), using a pTRE-tight-*hmi-er1α* construct. Control cell lines were generated by transfection with the pTRE-tight empty vector. Stable clones were induced to express MI-ER1*α* using 2 *μ*g ml^−1^ doxycycline (dox) and expression was verified by western blot analysis of whole cell extracts, using our anti-MI-ER1*α*-specific antibody (Paterno *et al*, 2002).

### Colony formation in soft agar

A total of 2 × 10^4^ cells were plated in 0.35% agarose on a layer of 0.5% agarose, prepared ±2 *μ*g ml^−1^ dox, ±10 nM E2; controls included the equivalent volume of buffer and/or ethanol. Media, dox and E2 were replenished every 4 days and colonies were stained with crystal violet after 18 days and counted.

### Study subjects

This study was approved by the Human Investigations Committee at Memorial University (HIC approval no. 05.56). One hundred and ten cases of primary invasive ductal carcinoma were identified from the database of the NL Eastern Health Cancer Registry for the years 2005 and 2006. Hematoxylin-and–eosin-stained slides for each case were reviewed by a pathologist (B. Carter) and all well-fixed tumour samples that contained adjacent normal ductal epithelium were selected for this study. One formalin-fixed, paraffin-embedded tissue block from each case was retrieved from the archives of the Department of Pathology and Laboratory Medicine, Memorial University, upon approval from Eastern Health's Medical Advisory and Board of Trustees Research Proposal Approval Committee.

### Tissue microarrays, whole tissue sections and immunohistochemistry

The MI-ER1*α* protein expression pattern was examined both in TMAs and in whole tissue sections. The tissue microarrays (TMAs) enabled us to compare a large number of samples under identical staining conditions, whereas whole tissue sections allowed us to examine larger areas of tumour and normal tissue from a single sample.

Sections of human adrenal gland and small intestine were purchased from US Biomax Inc. (Rockville, MD, USA). Microarrays were purchased from US Biomax Inc., Biochain (Hayward, CA, USA) and the Cooperative Human Tissue Network (http://chtn.nci.nih.gov). Fourteen different TMAs containing a total of 204 normal, 91 hyperplasia, 78 DCIS, 102 ILC and 343 IDC samples were stained. Any cores that were of questionable pathology were reviewed by a pathologist. Some of the samples could not be assessed as either the core was missing or contained insufficient relevant tissue. The number of cases that were scored in each category is listed in the Results section.

Immunohistochemistry and preabsorption with peptide was performed as described previously (Thorne *et al*, 2008) using the Universal LSAB^+^-HRP kit (Dako, Denmark) and 1.25 *μ*g ml^−1^ of anti-MI-ER1*α* IgG or pre-immune IgG. Antigen retrieval was performed in 10 mM sodium citrate, pH 6.0, in a 95 °C water bath. Optimum retrieval time was determined empirically to be 40 min for whole tissue sections and 30 min for TMAs. As a control, each batch included slides stained with pre-immune IgG. Each sample was evaluated for staining using the Allred scoring system (Allred *et al*, 1993; Harvey *et al*, 1999), which consists of a combined score for staining intensity (0=no, 1=weak, 2=moderate and 3=intense staining) and proportion of positive cells (0=0, 1=<1/100, 2=1/100–1/10, 3=1/10–1/3, 4=1/3–2/3, 5=>2/3 stained). The sum of the two produces an Allred score; the value for the tumour cells was compared with that of normal adjacent tissue. For the assessment of nuclear staining, samples were scored positive if at least 5% of the nuclei in the section were stained; however, the majority of normal samples that scored positive contained at least 50% positive nuclei, whereas the majority of positive tumour samples contained fewer than 10% positive nuclei.

### Statistical analysis

Correlation between the relative intensity of MI-ER1*α* staining in tumour sections and various clinicopathological parameters was subjected to *χ*^2^ analysis (Pearson's test, two-tailed, 95% confidence interval) using SPSS v.13.0 software. The percentage of nuclear staining in the carcinoma samples was compared with that in normal breast tissue using a two-sided Fisher's exact test and the InStat v.3.0 software program (GraphPad Software, San Diego, CA, USA).

## Results

### MI-ER1*α* interacts with ER*α in vivo*

**Figure 1:**
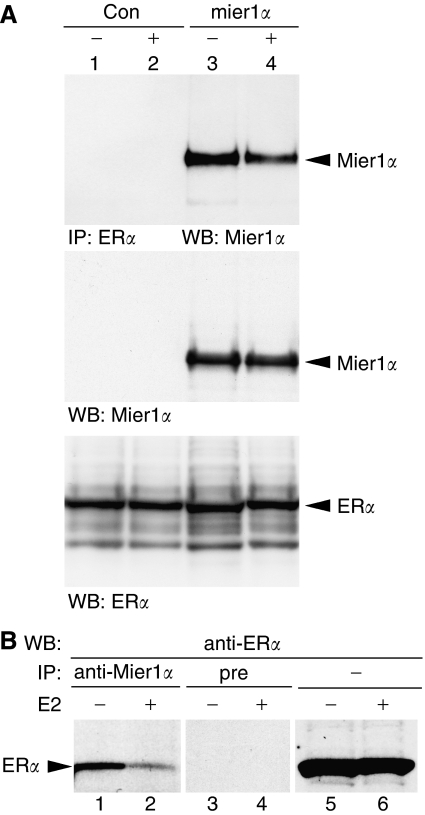
MI-ER1*α* interacts with ER*α in vivo*. (**A**) HEK293 cells were transfected with pcDNA3-*herα* and either pCS3+MT-*mier1α* (lanes 3 and 4) or control empty vector (lanes 1 and 2) and treated with vehicle (lanes1 and 3) or 10 nM E2 (lanes 2 and 4) for 3 h before extraction. Extracts were subjected to immunoprecipitation with anti-ER*α* (top panel) or loaded directly onto the gel (middle and bottom panels). Western blots were stained for MI-ER1*α* (top and middle panels) or ER*α* (bottom panel). (**B**) Extracts from MCF-7 cells treated with vehicle (lanes 1, 3 and 5) or 10 nM E2 (lanes 2, 4 and 6) were subjected to IP with anti-mier1*α* (lanes 1 and 2), pre-immune (lanes 3 and 4) or loaded directly onto the gel (lanes 5 and 6); Western blotting was performed with anti-ER*α*.

We investigated the ability of MI-ER1*α* to physically associate with ER*α* in HEK293 cells transfected with *mi-er1α* and *erα*, using co-IP assays. Our results show that ER*α* co-immunoprecipitates with MI-ER1*α* both in the presence and absence of ligand (E2); however, the intensity of the interaction was slightly reduced in the presence of ligand (Figure 1A). This difference was not due to variability in the expression levels of MI-ER1*α* or ER*α*, as these remained constant (Figure 1A).

To verify that endogenous MI-ER1*α* interacts with endogenous ER*α*, co-IP analysis of extracts from an ER+ breast carcinoma cell line, MCF-7, was performed using our anti-MI-ER1*α* antibody. As can be seen in Figure 1B, ER*α* was detected in the MI-ER1*α* immunoprecipitate (lanes 1 and 2), but not in the pre-immune immunoprecipitate (lanes 3 and 4), demonstrating a specific interaction between MI-ER1*α* and ER*α*. As seen in the HEK293 cells, this interaction was stronger in the absence of E2 (Figure 1B, lanes 1 and 2). These results demonstrate that endogenous complexes containing MI-ER1*α* and ER*α* exist in the cell.

### MI-ER1*α* reduces E2-stimulated growth of ER+ breast carcinoma cells

**Figure 2:**
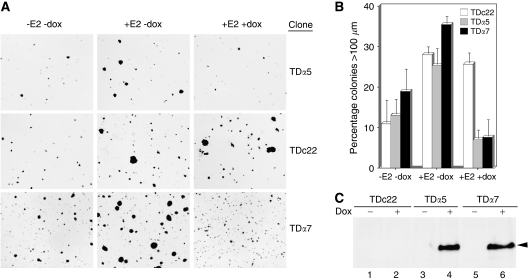
Overexpression of MI-ER1*α* reduces anchorage-independent growth of T47D breast carcinoma cells. Control (TDc22)- or MI-ER1*α* (TD*α*5 and TD*α*7)-expressing Tet-On T47D clones were cultured in 0.35% agarose, in the presence or absence of 2 *μ*g ml^−1^ dox and in the presence or absence of 10 nM E2, as described in the Materials and methods. Colonies were stained with crystal violet, and colony size was measured using an ocular micrometre. A minimum of six fields from each plate was analysed; the number of colonies larger than 100 *μ*m in size, expressed as a percentage of the total number of colonies, was recorded for each treatment. (**A**) Representative fields for each treatment combination for each clone are shown. (**B**) Histogram showing the average values and error bars for three independent experiments, performed in duplicate. (**C**) Western blot analysis to verify dox-specific induction of MI-ER1*α* expression. A representative blot of extracts from TDc22, TD*α*5 and TD*α*7 cells, cultured in the absence (lanes 1, 3 and 5) or presence (lanes 2, 4 and 6) of 2 *μ*g ml^−1^ dox, is shown. The position of MI-ER1*α* is indicated.

To investigate the functional effect of MI-ER1*α*-ER*α* interaction on cellular responses to E2, we produced dox-inducible MI-ER*α* T47D clonal cell lines. Control (TDc22) and MI-ER1*α*-expressing (TD*α*5 and TD*α*7) clones were assessed for their ability to proliferate in soft agar, when stimulated by E2. In the absence of dox, E2 stimulated the growth of both control and MI-ER1*α* clones, as measured by the increase in colony diameter (Figure 2A and B). However, induction of MI-ER1*α* expression by exposure to 2 *μ*g ml^−1^ dox dramatically reduced the ability of E2 to stimulate colony growth, although having no effect on the growth of TDc22 control cells (Figure 2A and B). Dox induction of MI-ER1*α* expression in the clones was verified by western blot analysis (Figure 2C). These results demonstrate that MI-ER1*α* reduces E2-stimulated anchorage-independent growth of breast carcinoma cells.

### Immunohistochemical analysis of MI-ER1*α* in normal breast and breast carcinoma

**Figure 3:**
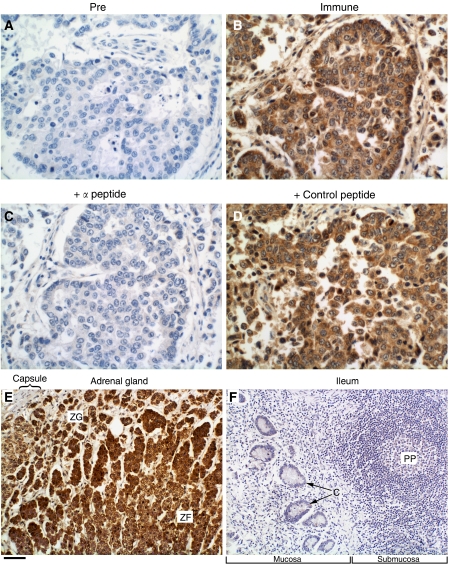
Immunohistochemical staining with anti-MI-ER1*α*. Human breast tumour sections were stained with pre-immune IgG (**A**), anti-mier1*α* IgG (**B**) or anti-mier1*α* IgG that had been pre-incubated with the *α*-specific peptide (**C**) or a control peptide (**D**). Note that only the peptide used to generate the antibody blocks staining. Sections of normal human adrenal gland (**E**) and normal human small intestine (**F**) stained with anti-mier1*α* IgG served as positive and negative tissue controls, respectively. Panel **E** shows a portion of the adrenal cortex that includes part of the zona glomerulosa (ZG) and zona fasciculata (ZF), whereas Panel **F** shows a section through the ileum that includes lymphoid tissue (Peyer's patch (PP)) and crypts of Lieberkühn (**C**). Scale bar=50 *μ*m for **A**–**D** and 100 *μ*m for **E** and **F**.

The MI-ER1*α* antibody used in this study has been well characterised and shown to be specific for the MI-ER1*α* protein (Paterno *et al*, 2002; Thorne *et al*, 2008). For this study, we used purified anti-MI-ER1*α* IgG and performed initial tests to confirm the specificity of antibody staining on paraffin sections of human tissue. Sections of breast tumours were stained in parallel with pre-immune IgG (Figure 3A), anti-MI-ER1*α* IgG (Figure 3B), anti-MI-ER1*α* IgG, which had been preabsorbed with the *α*-peptide used to generate the antibody (Figure 3C) or with an unrelated peptide (control peptide; Figure 3D). As can be seen in Figure 3, preabsorption of the antibody with the *α*-peptide blocked staining and produced results very similar to that obtained with pre-immune IgG. The control peptide, on the other hand, did not affect antibody binding, and the staining was similar to that obtained with the anti-MI-ER1*α* IgG alone. Additional positive and negative controls included sections of normal human adrenal cortex and small intestine, respectively; previously, we reported positive staining in the murine adrenal gland, whereas most areas of the small intestine were negative (Thorne *et al*, 2008). As can be seen in Figure 3E and F, staining of the human tissues was similar to that seen in the mouse, with intense staining of the adrenal cortex and no immunoreactivity in the ileum.

Of the TMAs used in this study, the cores that contained sufficient tissue for assessment included 180 cases of normal breast, 81 hyperplasia, 71 DCIS, 325 IDC and 99 ILC. In addition, there were 16 cores containing lymph node metastases. An additional 85 cases of IDC and adjacent normal breast tissue were examined in whole tissue sections.

**Figure 4:**
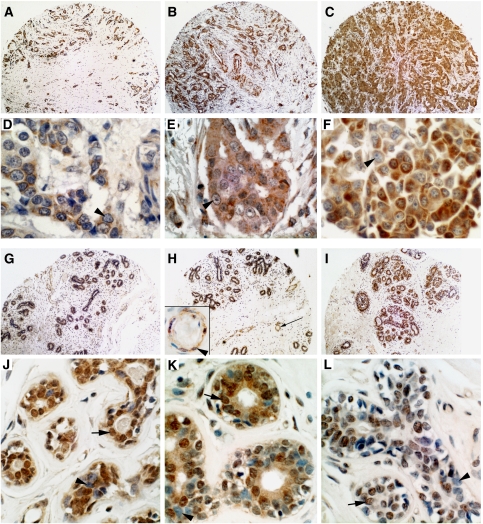
Expression pattern of MI-ER1*α* in normal breast tissue and invasive ductal carcinoma. (**A**–**C**) Low magnification of representative examples of primary invasive ductal carcinoma. (**D**–**F**) High magnification of IDC illustrating the subcellular localisation of MI-ER1*α*. Note that the nuclei (arrowheads) are negative (blue) and that MI-ER1*α* expression (brown) is exclusively cytoplasmic. (**G**–**I**) Low magnification of representative examples of normal breast tissue. MI-ER1*α* expression is detected primarily in the ducts with little or no expression in the surrounding stroma. The inset in H shows a higher magnification of the blood vessel indicated by a long arrow and illustrates weak staining of endothelial cells (arrowhead). (**J**–**L**) High magnification of normal ducts. Arrows indicate nuclear MI-ER1*α* staining; arrowheads indicate examples of negative nuclei. Scale bar=250 *μ*m for **A**–**C** and **G**–**I**, 25 *μ*m for **D**–**F**, **J**–**L** and inset in **H**.

First, we determined the MI-ER1*α* expression pattern. In normal breast tissue, staining was observed primarily in the ductal epithelial cells (Figure 4G–I); there was some weaker staining of vascular endothelial cells (inset in Figure 4H) but very little or no staining of the stroma. Some variability was observed in the intensity of staining of the ductal epithelium and no obvious difference in staining intensity of the ducts *vs* the lobules. In the tumours, the staining pattern was similar to that in normal tissue, with expression in the tumour cells themselves and little or no staining of the stroma (Figure 4A–C).

Next, staining in whole tissue sections was assessed using the Allred scoring system, which consists of a combined score for intensity level (0–3) and proportion of positive cells (0–5). The Allred score for the tumour was compared with that for adjacent normal tissue. Overall, there was no consistent difference in scores between the tumour and adjacent normal tissue, that is, some tumours had lower values than adjacent normal, some had higher values and some had equal values. These three categories of relative Allred scores were analysed for correlation with a number of clinical parameters, which included patient age, tumour size, lymph node status, grade (modified Scarff–Bloom–Richardson), stage (TNM), ER, PR and Her2/neu status (Table 1). No statistically significant correlation was found, as determined by *χ*^2^ analysis (Table 1). In addition, we analysed the intensity and proportion scores separately and found no statistically significant correlation (data not shown).

**Figure 5:**
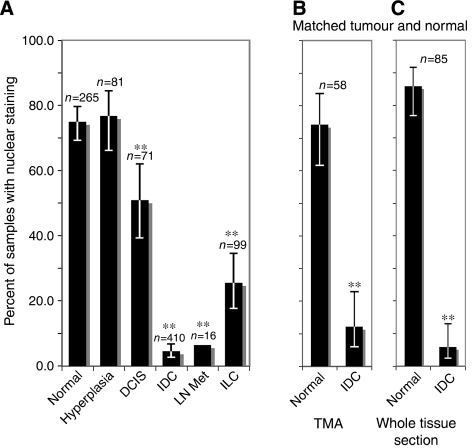
Loss of nuclear MI-ER1*α* during breast cancer progression. (**A**) Values from TMAs and whole tissue sections. The percentages and 95% confidence intervals are shown. The number of samples (*n*) in each category is listed above the bar; ^**^ indicates *P*⩽0.0001. (**B**) Values from TMAs containing a total of 58 matched normal and IDC samples. (**C**) Values from whole tissue sections; 85 samples containing matched IDC and normal adjacent tissue were analysed.

Samples were also examined for the subcellular localisation of MI-ER1*α* and initially scored as nuclear only, cytoplasmic only or nuclear and cytoplasmic. As virtually all samples had some cytoplasmic staining but the presence of nuclear staining was limited, samples were then separated into two categories: nuclear staining and no nuclear staining. The results were quite striking, showing a large differential between the percentage of normal tissue samples and IDC that were positive for nuclear staining: 74.7 *vs* 4.4%, respectively (*P*<0.0001; Figures 4D–F, 4J–L, 5A). In addition, the proportion of stained nuclei within a sample was different: most tumour samples that displayed nuclear MI-ER1*α* contained fewer than 10% stained nuclei, whereas the majority of normal samples contained >50% stained nuclei. A differential in nuclear staining between normal and ILC was observed as well (74.7 *vs* 25.3%; *P*<0.0001; Figure 4A). We examined nuclear staining in other subtypes, namely hyperplasia and DCIS. The percentage nuclear staining in hyperplasia was similar to that of normal samples (76.5%; *P*=0.8831; Figure 5A), whereas DCIS was intermediate between normal and IDC (50.7%; *P*=0.0001; Figure 5A). Cases of lymph node metastases from patients with IDC showed levels of nuclear staining similar to IDC (6.3%; *P*=0.5009; Figure 5A).

It was important to compare nuclear staining in tumour samples with adjacent normal tissue from the same patient, to ensure that the observed difference in MI-ER1*α* subcellular localisation is not merely due to individual patient differences. Therefore, we analysed 58 cases from TMAs that contained matched tumour–normal cores (Figure 5B); in addition, we compared tumour and normal adjacent areas in whole tissue sections from 85 cases of IDC (Figure 5C). Both gave results similar to that described above: the majority of tumours lacked nuclear staining, whereas most of the adjacent normal areas showed nuclear staining (Figure 5B and C). Together, these data demonstrate that a shift in the subcellular localisation of MI-ER1*α* is associated with breast cancer progression.

## Discussion

Our demonstration that MI-ER1*α* can physically interact with ER*α* and that regulated overexpression of MI-ER1*α* dramatically affects oestrogen-stimulated growth of breast carcinoma cells indicates that MI-ER1*α* functions as an ER corepressor. A number of ER*α* corepressors have been identified (Hall and McDonnell, 2005) and MI-ER1 is similar in structure and function to other known NR corepressors, including NCoR (Horlein *et al*, 1995), SMRT (Chen and Evans, 1995) and MTA1–3 (Manavathi and Kumar, 2007), which also recruit HDAC activity to repress transcription.

Traditionally, corepressors bind unliganded NRs, whereas coactivators bind liganded NRs; however, ER*α* is distinct from other NRs in this regard (Dobrzycka *et al*, 2003). For example, the classic corepressors, NCoR (Horlein *et al*, 1995) and SMRT (Chen and Evans, 1995), interact only with antagonist-bound ER*α*, whereas LCoR interacts with ER*α* only in the presence of oestrogen (Fernandes *et al*, 2003). Our results show that MI-ER1*α* binds ER*α* in the presence and absence of oestrogen and, in this regard, is similar to BRCA1, which regulates both oestrogen-stimulated and unliganded ER*α* activity (Fan *et al*, 2001; Rosen *et al*, 2005).

Previously, we reported a PCR analysis that demonstrated a significant increase in *mi*-*er1* mRNA expression levels in breast tumours when compared with normal breast tissue (Paterno *et al*, 1998). However, our current study shows no consistent difference in MI-ER1*α* protein expression levels. This discrepancy is most likely due to the fact that MI-ER1*α* expression in normal tissue is restricted primarily to ductal epithelial cells, whereas within the tumour, virtually all tumour cells are positive for MI-ER1*α*. Thus, the proportion of cells expressing *mi-er1* mRNA per volume of tissue would be much lower in normal breast than in a solid tumour sample.

Our immunohistochemical analysis showed that although virtually all (96%) IDC samples have lost nuclear MI-ER1*α*, only half of the DCIS samples showed this shift in subcellular localisation. Ductal carcinoma *in situ* consists of a heterogeneous group of pre-invasive lesions that may or may not progress to IDC and, at the moment, it is not possible to accurately predict which patients are at risk (Wiechmann and Kuerer, 2008). Thus, it will be important to determine the value of MI-ER1*α* as a prognostic indicator, using a larger sample size. A key question is whether loss of nuclear MI-ER1*α* is associated with specific subtypes of DCIS or any other parameter correlated with progression to IDC. Such parameters include histological criteria, for example, nuclear grade and cell necrosis, as well as molecular markers, for example, loss of IGFBP-rP1 expression (Wiechmann and Kuerer, 2008). As more prognostic markers are identified, it may become possible to define a molecular-histological fingerprint for DCIS that can accurately predict a patient's risk of recurrence as invasive carcinoma.

Our observations in this study are consistent with a model where, in the progression to IDC, MI-ER1*α* is shuttled to the cytoplasm such that it cannot exert its gene/chromatin repressor functions. This may include loss of the functional interaction between MI-ER1*α* and ER*α*. Alternatively, it may act in a manner similar to MTA1s, a splicing isoform of MTA1, which binds to ER*α* in the cytoplasm and enhances ER*α* non-genomic activities, including stimulation of peptide growth factor signal transduction pathways (Kumar *et al*, 2002). The net result is an increase in breast carcinoma cell proliferation. We are currently investigating the molecular mechanisms of MI-ER1*α* localisation in breast cancer cells and its role in tumour progression to an invasive phenotype.

## Figures and Tables

**Table 1:** Analysis of correlation between clinicopathological parameters and relative MIER1*α* staining in breast carcinoma

	**MIER1 expression:^a^ tumor Allred score *vs* normal Allred score**				
**Parameters**	**Less than normal**	**Equal to normal**	**Greater than normal**	**Total**	***P*-value^b^**
*Age*					
<50	6 (19.4)	4 (12.9)	21 (67.7)	**31**	
>=50	8 (10.1)	16 (20.3)	55 (69.6)	**79**	*P*=0.410
					
*Age*					
<=44	5 (25.0)	3 (15.0)	12 (60.0)	**20**	
44–64	4 (7.5)	9 (17.0)	40 (75.5)	**53**	
>=65	5 (13.9)	8 (19.4)	24 (66.7)	**37**	*P*=0.365
					
*Tumor size (TNM)*					
T1	6 (10.2)	11 (18.6)	42 (71.2)	**59**	
T2	7 (16.7)	6 (14.3)	29 (69.0)	**42**	
T3 and T4	1 (11.1)	3 (33.3)	5 (55.6)	**9**	*P*=0.618
					
*Tumor size (categorical)*					
<=2 cm	6 (10.2)	11 (18.6)	42 (71.2)	**59**	
>2 cm	8 (15.7)	9 (17.6)	34 (66.7)	**51**	*P*=0.693
					
*Lymph node involvement*					
No	6 (13.6)	9 (20.5)	28 (63.6)	**43**	
Yes	4 (12.5)	6 (18.8)	22 (68.8)	**32**	*P*=0.957
					
*Lymph node status (TNM)*					
N0	6 (13.6)	9 (20.5)	28 (63.6)	**43**	
N1	4 (16.0)	5 (20.0)	16 (64.0)	**25**	
N2	0 (0)	0 (0)	6 (100.0)	**6**	
N3	0 (0)	0 (0)	1 (100.0)	**1**	*P*=0.941
					
*Grade* c					
1	6 (20.0)	8 (26.7)	16 (53.3)	**30**	
2	5 (11.9)	8 (19.1)	29 (69.0)	**42**	
3	3 (8.1)	4 (10.8)	30 (81.1)	**37**	*P*=0.217
					
*Stage* d					
I	1 (4.0)	6 (24.0)	18 (72.0)	**25**	
II	9 (23.1)	4 (10.3)	26 (66.6)	**39**	
III	0 (0)	3 (27.3)	8 (72.7)	**11**	
IV	0	0	0	**0**	*P*=0.082
					
*ER*					
Negative	2 (12.5)	2 (12.5)	12 (75.0)	**16**	
Positive	10 (11.2)	16 (18.0)	63 (70.8)	**89**	*P*=0.891
					
*PR*					
Negative	3 (10.8)	2 (7.1)	23 (82.1)	**28**	
Positive	9 (11.8)	15 (19.7)	52 (68.4)	**76**	*P*=0.300
					
*HER2/neu status*					
Negative	10 (13.5)	15 (20.3)	49 (66.2)	**74**	
Positive	1 (6.7)	0 (0)	14 (93.3)	**15**	*P*=0.086

a Values are listed as number of subjects, with percentage listed in brackets.

b *P*-value; *χ*^2^ analysis (Pearson's test, two-tailed, 95% CI), statistical significance is assumed when *P*<0.05.

c Modified Scarff–Bloom–Richardson grading system.

d FIGO staging system.
